# Stochastic Delay Modelling of Streptococcal Transmission: Stability Analysis and Dynamically Consistent Numerical Schemes

**DOI:** 10.1049/syb2.70086

**Published:** 2026-10-03

**Authors:** Ali Raza, Mansoor Alsulami, Emad Fadhal, Faridah Alruwaili

**Affiliations:** ^1^ Department of Mathematics Fırat University Elazığ Türkiye; ^2^ Department of Mathematics Faculty of Science King Abdulaziz University Jeddah Saudi Arabia; ^3^ Department of Mathematics and Statistics College of Science King Faisal University Al Ahsa Saudi Arabia; ^4^ Department of Mathematical Sciences College of Science Princess Nourah Bint Abdulrahman University Riyadh Saudi Arabia

**Keywords:** computational complexity, control nonlinearities, convergence of numerical methods, difference equations, diseases, stochastic systems

## Abstract

This study investigates the transmission dynamics of streptococcal disease using a stochastic delay differential equation (SDDE) framework. The population is divided into four compartments: susceptible individuals S, infected individuals I, carriers of *streptococcus* C and individuals who progress to the post‐infectious complication of acute rheumatic fever (ARF), representing severe disease outcomes rather than an infectious transmission class, A. The incorporated discrete time delay represents the effective latency associated with bacterial colonisation, disease incubation and the early host immune response prior to disease progression. The deterministic delayed model is first analysed to establish fundamental dynamical properties, including positivity, boundedness and the existence and uniqueness of solutions. The equilibrium states are derived and their local and global stability are examined in relation to the basic reproduction number, $R_{0}$, which serves as the epidemiological threshold determining whether the disease dies out or persists within the population. A stochastic delayed version of the model is then formulated, and conditions ensuring the existence of a unique global positive solution are obtained. Furthermore, a dynamically consistent stochastic nonstandard finite difference (NSFD) scheme is constructed by combining the nonstandard finite difference methodology with an Euler–Maruyama discretisation of the stochastic perturbation terms to preserve the qualitative dynamics of the continuous SDDE model. The proposed scheme preserves essential dynamical properties of the system and is shown to converge to the corresponding equilibrium states without restrictions on the step size. Numerical simulations are carried out to compare the proposed scheme with several standard stochastic numerical methods. The results demonstrate that the nonstandard finite difference scheme maintains positivity and boundedness of the solutions and closely follows the mean trajectory of the deterministic model, whereas the standard schemes may violate these properties. The proposed framework therefore provides a reliable and dynamically consistent computational tool for investigating the influence of stochastic perturbations and delayed disease progression on streptococcal transmission dynamics. Finally, the influence of stochasticity and time delay on the dynamics of susceptible and infected populations is illustrated through numerical experiments.

## Graphical abstract

This study develops and analyses a stochastic delay differential equation model for the transmission dynamics of streptococcal disease, incorporating susceptible, infected, carrier and acute rheumatic fever compartments. The model's qualitative properties, including existence, positivity and stability, are established, and a stochastic nonstandard finite difference scheme is proposed for reliable numerical approximation. Numerical results demonstrate that the scheme preserves key dynamical features and effectively captures the effects of stochasticity and time delay on disease dynamics.

## 1 Introduction

Group A *Streptococcus* (GAS), also known as *Streptococcus pyogenes*, is a bacterial pathogen responsible for a variety of infectious diseases. These infections include pharyngitis, tonsillitis, scarlet fever, impetigo, cellulitis and other skin and soft tissue infections. The first detailed description of streptococcal infection was given by the Austrian surgeon Theodor Billroth in 1874 while studying cases of erysipelas and wound infections. GAS spreads mainly through respiratory droplets produced during coughing or sneezing, as well as through direct contact with contaminated surfaces or infected skin.

In most cases, GAS infections can be effectively treated with commonly available antibiotics such as penicillin. For individuals allergic to penicillin, erythromycin may be used as an alternative treatment, although resistance has occasionally been reported. Despite the availability of treatment, untreated or improperly treated GAS infections may lead to serious complications, particularly acute rheumatic fever (ARF). ARF primarily affects children between the ages of 5 and 15 and may cause severe health problems such as joint inflammation, skin manifestations, neurological disorders and permanent heart damage. Epidemiological studies suggest that approximately 0.3%–3% of GAS infections progress to ARF, and individuals with a previous history of ARF have a significantly higher risk of recurrence.

Mathematical modelling has become an essential tool for understanding the transmission dynamics of infectious diseases and for evaluating possible control strategies. Various mathematical models have been developed to investigate pneumococcal and streptococcal infections. For example, Zephaniah et al. [1] analysed an SVEIR model for *Streptococcus pneumoniae* and showed that the disease dies out when the basic reproduction number $R_{0}<1$, whereas persistence occurs when $R_{0}>1$. Coen et al. [2] studied the transmission dynamics of meningococcal infection using epidemiological data from Belgian schoolchildren and estimated the average duration of bacterial carriage. Patanarapeelert et al. [3] investigated deterministic and stochastic models describing the progression of streptococcal infections leading to acute rheumatic fever.

Several studies have also examined the effects of treatment, vaccination and antibiotic resistance on pneumococcal infections. Opatowski et al. [4] analysed how antibiotic dosage influences resistance development in *S. pneumoniae*. Kizito and Tumwiine [5] developed a mathematical model incorporating vaccination and treatment interventions, showing that effective control strategies can lead to global disease elimination. Houben et al. [6] used mathematical modelling to study the transmission dynamics of *Streptococcus equi* and highlighted the importance of carrier detection and vaccination in disease control.

Other works have explored different epidemiological aspects of pneumococcal disease. Bottomley et al. [7] studied serotype replacement following vaccination, whereas Smith et al. [8] modelled the immune response to pneumococcal lung infection. Stochastic approaches have also been widely applied in epidemiological modelling to account for environmental variability and random effects in disease transmission [9]. These models often provide a more realistic description of infectious disease dynamics compared to purely deterministic formulations. Recently, dynamically consistent numerical schemes have attracted considerable attention for solving stochastic epidemic models while preserving important qualitative properties such as positivity, boundedness and stability. Robust NSFD approaches have been successfully developed for stochastic epidemic systems with treatment and partial immunity [10], stochastic reaction–diffusion chemical models [11] and stochastic SIRS epidemic models with partial immunity and nonlinear incidence [12]. Motivated by these advances, the present work extends the NSFD methodology to a stochastic delayed streptococcal transmission model, providing a reliable computational framework that preserves the essential dynamics of the underlying continuous system.

Despite significant progress in mathematical modelling of streptococcal infections, several aspects of disease transmission and progression remain insufficiently understood, particularly when stochastic effects and time delays are incorporated into the model. Time delays may represent incubation periods, treatment delays or other biological processes influencing disease progression. Incorporating such delays into stochastic models can provide a more realistic representation of infection dynamics.

Motivated by these considerations, the present study develops and analyses a stochastic delayed epidemic model for the transmission dynamics of streptococcal disease. The model incorporates both deterministic and stochastic components to better capture the uncertainty present in real epidemiological systems.

Unlike existing deterministic and conventional stochastic models for streptococcal disease transmission, the present study integrates stochastic perturbations with discrete time delays within a unified modelling framework. The principal contributions include the theoretical analysis of the stochastic delayed system, the establishment of existence, uniqueness, positivity and stability results, and the development of a dynamically consistent stochastic nonstandard finite difference scheme that preserves the qualitative properties of the continuous model for arbitrary time step sizes. These features distinguish the proposed framework from previously published deterministic, fractional‐order and standard stochastic numerical approaches.

The numerical results are compared with those obtained using conventional stochastic numerical methods, demonstrating the improved qualitative performance and dynamical consistency of the proposed stochastic NSFD scheme.

The principal contributions of this study are threefold. First, a stochastic delayed model for streptococcal disease transmission is formulated by incorporating biologically motivated time delays and environmental stochastic perturbations. Second, rigorous theoretical results are established concerning the existence, uniqueness, positivity and stability of the proposed stochastic delayed system. Third, a dynamically consistent stochastic nonstandard finite difference scheme is developed and shown to preserve the essential qualitative properties of the continuous model while providing reliable numerical approximations for long‐term simulations. The remainder of the paper is organised as follows. Section 2 presents the formulation of the delayed deterministic model, establishes its fundamental properties, and investigates the equilibrium states and stability of the system. Section 3 presents the stochastic delayed model, establishes the existence and uniqueness of a global positive solution, and describes the numerical procedure used to solve the model. Section 4 provides numerical simulations illustrating the theoretical results. Section 5 concludes the paper with a summary of the main findings.

## 2 Derivation of the Delayed Streptococcal Mathematical Model

The deterministic compartmental model considered in this study is adapted from previously published mathematical models of streptococcal disease transmission [4, 5, 6]. The novelty of the present work lies in extending these deterministic formulations by incorporating a biologically motivated discrete time delay together with stochastic perturbations, thereby providing a more realistic representation of the transmission dynamics under environmental uncertainty.

The proposed model is based on the following biological assumptions:

1. The total human population is divided into four mutually exclusive compartments: susceptible individuals (S), symptomatic infected individuals (I), asymptomatic carriers (C) and individuals with post‐infectious acute rheumatic fever (A).
2. Recruitment into the susceptible population occurs at a constant rate Λ, whereas all individuals experience natural mortality at rate *µ*.
3. Disease transmission occurs through effective contact between susceptible individuals and both symptomatic infected individuals and asymptomatic carriers.
4. Acute rheumatic fever (ARF) is regarded as a post‐infectious autoimmune complication of Group A *Streptococcus* infection and therefore does not contribute to further disease transmission.
5. Environmental variability and other random effects influencing disease transmission are represented through stochastic perturbations.

Let N(t) denote the total human population at time t≥0. The population is divided into four epidemiological compartments: susceptible individuals S(t), infected individuals I(t), carriers of *streptococcus* C(t) and individuals who develop acute rheumatic fever A(t). Thus,

N(t)=S(t)+I(t)+C(t)+A(t).

To describe the transmission process more realistically, we employ a saturated incidence function as proposed in Ref. [13]. A single discrete time delay τ is incorporated to represent the effective biological latency associated with bacterial colonisation, disease incubation and the early host immune response before infected individuals contribute fully to disease progression. Although the natural history of Group A *Streptococcus* involves several clinically distinct time intervals, the present study adopts a single effective delay to maintain mathematical tractability and because reliable epidemiological data for estimating multiple independent delays are not available.

The delayed transmission term is further multiplied by the survival factor $e^{-\mu \tau }$, which accounts for the probability that individuals survive natural mortality during the delay interval before becoming infectious. Consequently, the delayed infection term models the transition of susceptible individuals to the infected class while incorporating both biological latency and demographic survival during the delay period.

The structure of the proposed model and the flow of individuals between epidemiological compartments are illustrated in Figure 1, whereas the corresponding model variables, parameters and their biological descriptions are summarised in Table 1.

**FIGURE 1:**
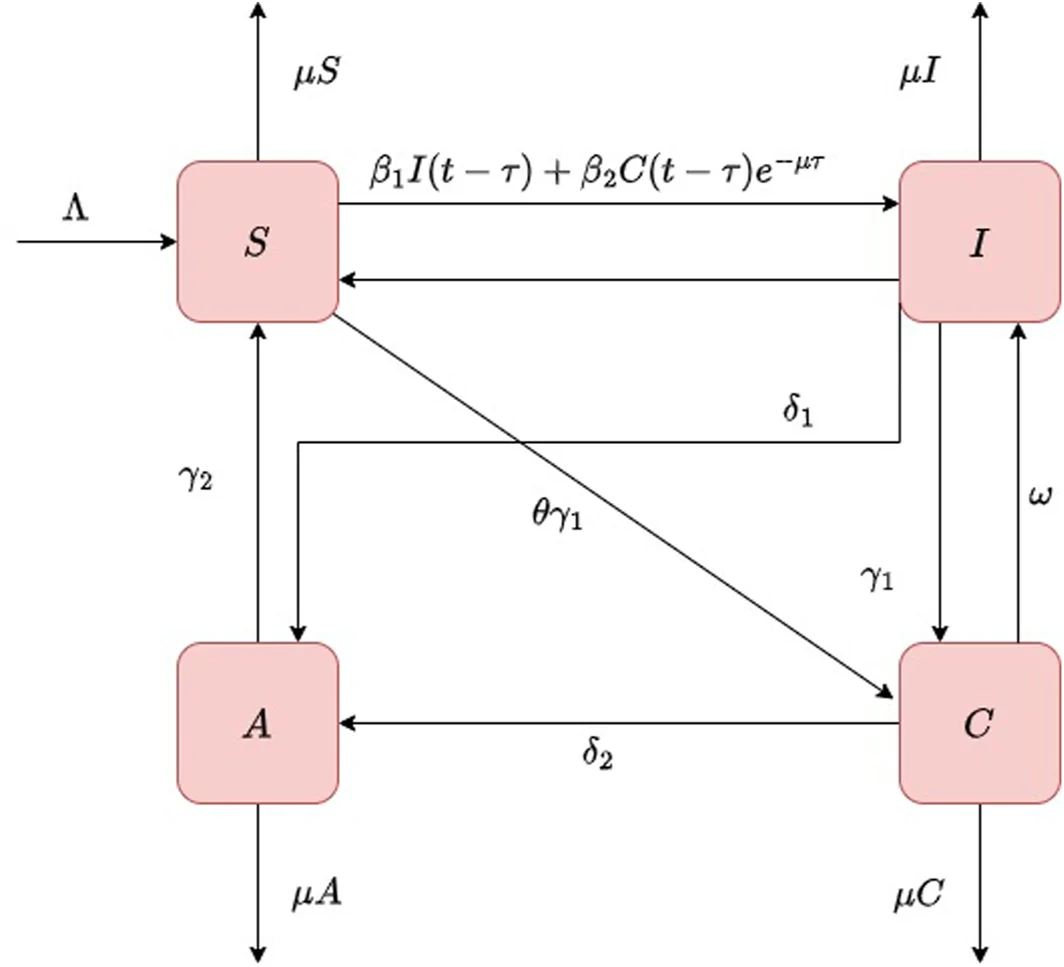
Flow chart of the delayed streptococcal epidemic model.

**TABLE 1:** Description of the model variables, parameters and numerical values used in the disease‐free equilibrium (DFE) and disease‐persistent equilibrium (DPE) simulations.

Symbol	Description	DFE	DPE	Source
S(t)	Susceptible individuals	—	—	Model variable
I(t)	Symptomatic individuals infected with group A Streptococcus	—	—	Model variable
C(t)	Asymptomatic carriers of group A Streptococcus	—	—	Model variable
A(t)	Individuals with post‐infectious acute rheumatic fever (ARF)	—	—	Model variable
Λ	Recruitment rate into the susceptible population	0.5	0.5	Assumed
$\beta_{1}$	Transmission rate from symptomatic infected individuals	1.089	2.089	Estimated
$\beta_{2}$	Transmission rate from carrier individuals	1.05	2.05	Estimated
*µ*	Natural mortality rate	0.5	0.5	Assumed
$\gamma_{1}$	Treatment/recovery rate of infected individuals	0.01	0.01	Literature
θ	Effectiveness of antibiotic treatment	0.09	0.09	Literature
$\gamma_{2}$	Recovery rate of individuals with acute rheumatic fever	0.08	0.08	Literature
ω	Rate at which carrier individuals develop symptomatic infection	0.0027	0.0027	Estimated
$\delta_{1}$	Progression rate from infected individuals to ARF	0.2	0.2	Literature
$\delta_{2}$	Progression rate from carriers to ARF	0.1	0.1	Literature
$\sigma_{1}$	Noise intensity in the susceptible compartment	0.05	0.05	Assumed
$\sigma_{2}$	Noise intensity in the infected compartment	0.04	0.04	Assumed
$\sigma_{3}$	Noise intensity in the carrier compartment	0.03	0.03	Assumed
$\sigma_{4}$	Noise intensity in the ARF compartment	0.05	0.05	Assumed
τ	Effective biological time delay representing bacterial colonisation, incubation and early immune response	τ	τ	Assumed

The delayed streptococcal epidemic model is formulated as

(1) $$\left\{\begin{array}{cc} \frac{dS}{dt} & =\Lambda -\left(\beta_{1}I\left(t-\tau \right)+\beta_{2}C\left(t-\tau \right)\right)S\left(t-\tau \right)e^{-\mu \tau }+\gamma_{2}A+\theta \gamma_{1}I-\mu S,\\ \frac{dI}{dt} & =\left(\beta_{1}I\left(t-\tau \right)+\beta_{2}C\left(t-\tau \right)\right)S\left(t-\tau \right)e^{-\mu \tau }+\omega C-\left(\gamma_{1}+\delta_{1}+\mu \right)I,\\ \frac{dC}{dt} & =\left(1-\theta \right)\gamma_{1}I-\left(\omega +\delta_{2}+\mu \right)C,\\ \frac{dA}{dt} & =\delta_{1}I+\delta_{2}C-\left(\gamma_{2}+\mu \right)A. \end{array}\right.$$

The corresponding initial conditions are

S(ξ)≥0,I(ξ)≥0,C(ξ)≥0,A(ξ)≥0,ξ∈[−τ,0].

### 2.1 Positivity, Boundedness and Existence of Solutions

For the delayed system, we consider the initial history functions

$$S\left(\xi \right)=\phi_{1}\left(\xi \right)\geq 0,\;I\left(\xi \right)=\phi_{2}\left(\xi \right)\geq 0,\;C\left(\xi \right)=\phi_{3}\left(\xi \right)\geq 0,\;A\left(\xi \right)=\phi_{4}\left(\xi \right)\geq 0,\;\xi \in \left[-\tau ,0\right],$$

where $\phi_{i}\in C\left(\left[-\tau ,0\right],R_{+}\right)$, i=1,2,3,4.

The delayed streptococcal epidemic model is given by

(2) $$\left\{\begin{array}{ccc} \frac{dS\left(t\right)}{dt} & = & \Lambda -\left(\beta_{1}I\left(t-\tau \right)+\beta_{2}C\left(t-\tau \right)\right)S\left(t-\tau \right)e^{-\mu \tau }+\gamma_{2}A\left(t\right)+\theta \gamma_{1}I\left(t\right)-\mu S\left(t\right),\\ \frac{dI\left(t\right)}{dt} & = & \left(\beta_{1}I\left(t-\tau \right)+\beta_{2}C\left(t-\tau \right)\right)S\left(t-\tau \right)e^{-\mu \tau }+\omega C\left(t\right)-\left(\gamma_{1}+\delta_{1}+\mu \right)I\left(t\right),\\ \frac{dC\left(t\right)}{dt} & = & \left(1-\theta \right)\gamma_{1}I\left(t\right)-\left(\omega +\delta_{2}+\mu \right)C\left(t\right),\\ \frac{dA\left(t\right)}{dt} & = & \delta_{1}I\left(t\right)+\delta_{2}C\left(t\right)-\left(\gamma_{2}+\mu \right)A\left(t\right). \end{array}\right.$$

Theorem 1 Suppose that the initial functions satisfy

$$\phi_{i}\left(\xi \right)\geq 0,\;\xi \in \left[-\tau ,0\right],\;i=1,2,3,4.$$

Then, the solution (S(t),I(t),C(t),A(t)) of system Equation (2) remains nonnegative for all t≥0. In other words,

$$(S\left(t\right),I\left(t\right),C\left(t\right),A\left(t\right))\in R_{+}^{4},\;\forall t\geq 0.$$

*Proof*: Let $t_{0}>0$ be the first time at which one of the state variables reaches zero. Since the initial history is nonnegative, all state variables are nonnegative on $\left[-\tau ,t_{0}\right]$. We examine the derivative of each component at such a boundary point.

If $S\left(t_{0}\right)=0$, then from Equation (2),

$${\left.\frac{dS\left(t\right)}{dt}\right|}_{t=t_{0}}=\Lambda +\gamma_{2}A\left(t_{0}\right)+\theta \gamma_{1}I\left(t_{0}\right)\geq 0.$$

Hence, S(t) cannot cross the hyperplane S=0 from above to below.

If $I\left(t_{0}\right)=0$, then

$${\left.\frac{dI\left(t\right)}{dt}\right|}_{t=t_{0}}=\left(\beta_{1}I\left(t_{0}-\tau \right)+\beta_{2}C\left(t_{0}-\tau \right)\right)S\left(t_{0}-\tau \right)e^{-\mu \tau }+\omega C\left(t_{0}\right)\geq 0.$$

If $C\left(t_{0}\right)=0$, then

$${\left.\frac{dC\left(t\right)}{dt}\right|}_{t=t_{0}}=\left(1-\theta \right)\gamma_{1}I\left(t_{0}\right)\geq 0,$$

provided 0≤θ≤1.

If $A\left(t_{0}\right)=0$, then

$${\left.\frac{dA\left(t\right)}{dt}\right|}_{t=t_{0}}=\delta_{1}I\left(t_{0}\right)+\delta_{2}C\left(t_{0}\right)\geq 0.$$

Therefore, none of the solution components can become negative. Hence, the nonnegative cone $R_{+}^{4}$ is positively invariant, and the solution remains nonnegative for all t≥0.

Theorem 2 The closed set

$$\Omega =\left\{\left(S,I,C,A\right)\in R_{+}^{4}:S+I+C+A\leq \frac{\Lambda }{\mu }\right\}$$

is positively invariant and attracting for system Equation (2).

*Proof*: Let

N(t)=S(t)+I(t)+C(t)+A(t).

Adding the four equations in Equation (2), we obtain

$$\frac{dN\left(t\right)}{dt}=\Lambda -\mu S\left(t\right)-\mu I\left(t\right)-\mu C\left(t\right)-\mu A\left(t\right)=\Lambda -\mu N\left(t\right).$$

Thus,

$$\frac{dN\left(t\right)}{dt}+\mu N\left(t\right)=\Lambda .$$

Solving this linear differential equation gives

$$N\left(t\right)=N\left(0\right)e^{-\mu t}+\frac{\Lambda }{\mu }\left(1-e^{-\mu t}\right).$$

Hence,

$$N\left(t\right)\leq N\left(0\right)e^{-\mu t}+\frac{\Lambda }{\mu },\;t\geq 0,$$

and therefore

$${\mathrm{lim sup}}_{t\to \infty }N\left(t\right)\leq \frac{\Lambda }{\mu }.$$

Consequently, all solutions of Equation (2) eventually enter and remain in the region

$$\Omega =\left\{\left(S,I,C,A\right)\in R_{+}^{4}:S+I+C+A\leq \frac{\Lambda }{\mu }\right\}.$$

Thus, Ω is positively invariant and attracting.

Theorem 3 For any initial history

$$\Phi \left(\xi \right)=\left(\phi_{1}\left(\xi \right),\phi_{2}\left(\xi \right),\phi_{3}\left(\xi \right),\phi_{4}\left(\xi \right)\right)\in C\left(\left[-\tau ,0\right],R_{+}^{4}\right),$$

system Equation (2) admits a unique global solution

$$(S\left(t\right),I\left(t\right),C\left(t\right),A\left(t\right))\in C\left(\left[-\tau ,\infty \right),R_{+}^{4}\right).$$

*Proof*: Define

$$X\left(t\right)=\left(\begin{array}{c} S\left(t\right)\\ I\left(t\right)\\ C\left(t\right)\\ A\left(t\right) \end{array}\right),\;X_{t}\left(\xi \right)=X\left(t+\xi \right),\;\xi \in \left[-\tau ,0\right].$$

Then, system Equation (2) can be written in the functional form

$$X^{\prime }\left(t\right)=F\left(X_{t}\right),$$

where $F:C\left(\left[-\tau ,0\right],R^{4}\right)\to R^{4}$ is given by the right‐hand side of Equation (2).

Since each component of F is a polynomial combination of the present and delayed variables, the mapping F is continuous and locally Lipschitz on bounded subsets of $C\left(\left[-\tau ,0\right],R^{4}\right)$. Therefore, by the standard existence and uniqueness theorem for delay differential equations, there exists a unique local solution on some interval $\left.\left[-\tau ,T_{\max}\right.\right)$.

It remains to show that this solution is global. By Theorem 1, the solution remains nonnegative, and by Theorem 2,

$$0\leq S\left(t\right)+I\left(t\right)+C\left(t\right)+A\left(t\right)\leq \max\left\{N\left(0\right),\frac{\Lambda }{\mu }\right\},\;t\geq 0.$$

Hence, each component is bounded on every finite interval, so no blow‐up can occur in finite time. Therefore, the local solution extends to all t≥0.

Thus, system Equation (2) admits a unique global nonnegative solution for every nonnegative continuous initial history. □

### 2.2 Equilibrium States

The equilibrium points of system Equation (2) are obtained by setting

$$\frac{dS}{dt}=\frac{dI}{dt}=\frac{dC}{dt}=\frac{dA}{dt}=0.$$

Since an equilibrium is constant in time, we have

$$S\left(t-\tau \right)=S^{*},\;I\left(t-\tau \right)=I^{*},\;C\left(t-\tau \right)=C^{*},\;A\left(t-\tau \right)=A^{*}.$$

Thus, the steady‐state system becomes

(3) $$\left\{\begin{array}{cc} 0 & =\Lambda -\left(\beta_{1}I^{*}+\beta_{2}C^{*}\right)S^{*}e^{-\mu \tau }+\gamma_{2}A^{*}+\theta \gamma_{1}I^{*}-\mu S^{*},\\ 0 & =\left(\beta_{1}I^{*}+\beta_{2}C^{*}\right)S^{*}e^{-\mu \tau }+\omega C^{*}-\left(\gamma_{1}+\delta_{1}+\mu \right)I^{*},\\ 0 & =\left(1-\theta \right)\gamma_{1}I^{*}-\left(\omega +\delta_{2}+\mu \right)C^{*},\\ 0 & =\delta_{1}I^{*}+\delta_{2}C^{*}-\left(\gamma_{2}+\mu \right)A^{*}. \end{array}\right.$$

#### 2.2.1 Disease‐Free Equilibrium

The disease‐free equilibrium is obtained by setting

$$I^{*}=0,\;C^{*}=0,\;A^{*}=0.$$

Then, from the first equation of Equation (3), we get

$$\Lambda -\mu S^{*}=0,$$

which gives

$$S^{*}=\frac{\Lambda }{\mu }.$$

Hence, the disease‐free equilibrium is

(4) $$E_{0}=\left(\frac{\Lambda }{\mu },\;0,\;0,\;0\right).$$

#### 2.2.2 Endemic Equilibrium

The endemic equilibrium $E^{*}=\left(S^{*},I^{*},C^{*},A^{*}\right)$ exists when

$$I^{*}>0,\;C^{*}>0,\;A^{*}>0.$$

From the third equation of Equation (3), we obtain

(5) $$C^{*}=\frac{\left(1-\theta \right)\gamma_{1}}{\omega +\delta_{2}+\mu }\;I^{*}.$$

From the fourth equation, it follows that

(6) $$A^{*}=\frac{\delta_{1}I^{*}+\delta_{2}C^{*}}{\gamma_{2}+\mu }.$$

Substituting Equation (5) into the second equation of Equation (3), we get

$$\left(\beta_{1}I^{*}+\beta_{2}C^{*}\right)S^{*}e^{-\mu \tau }=\left(\gamma_{1}+\delta_{1}+\mu \right)I^{*}-\omega C^{*}.$$

Using Equation (5), this becomes

$$\left(\beta_{1}+\beta_{2}k\right)S^{*}e^{-\mu \tau }I^{*}=\left(\left(\gamma_{1}+\delta_{1}+\mu \right)-\omega k\right)I^{*},$$

where

$$k=\frac{\left(1-\theta \right)\gamma_{1}}{\omega +\delta_{2}+\mu }.$$

Since $I^{*}>0$, we obtain

(7) $$S^{*}=\frac{\left(\gamma_{1}+\delta_{1}+\mu \right)\left(\omega +\delta_{2}+\mu \right)-\omega \left(1-\theta \right)\gamma_{1}}{e^{-\mu \tau }\left[\beta_{1}\left(\omega +\delta_{2}+\mu \right)+\beta_{2}\left(1-\theta \right)\gamma_{1}\right]}.$$

Therefore, the endemic equilibrium can be written as

(8) $$E^{*}=\left(S^{*},I^{*},C^{*},A^{*}\right),$$

where $S^{*}$ is given by Equation (7),

$$C^{*}=\frac{\left(1-\theta \right)\gamma_{1}}{\omega +\delta_{2}+\mu }\;I^{*},$$

and

$$A^{*}=\frac{\delta_{1}I^{*}+\delta_{2}C^{*}}{\gamma_{2}+\mu }.$$

Moreover, since at equilibrium

$$N^{*}=S^{*}+I^{*}+C^{*}+A^{*}=\frac{\Lambda }{\mu },$$

we obtain

(9) $$I^{*}=\frac{\frac{\Lambda }{\mu }-S^{*}}{1+\frac{\left(1-\theta \right)\gamma_{1}}{\omega +\delta_{2}+\mu }+\frac{\delta_{1}+\delta_{2}\frac{\left(1-\theta \right)\gamma_{1}}{\omega +\delta_{2}+\mu }}{\gamma_{2}+\mu }}.$$

Hence, $C^{*}$ and $A^{*}$ follow directly from Equations (5) and (6).

### 2.3 Basic Reproduction Number

The basic reproduction number, denoted by $R_{0}$, is defined as the average number of secondary infections generated by a single infectious individual introduced into a wholly susceptible population. We compute $R_{0}$ using the next‐generation matrix approach [14].

The infected compartments of system Equation (2) are I, C, and A. However, new infections are produced only through the infected classes I and C. Thus, the new infection matrix and transition matrix are constructed from the subsystem associated with (I,C,A).

Let

$$X={\left(I,C,A\right)}^{T}.$$

Then, the infected subsystem can be written as

$$\frac{dX}{dt}=F\left(X\right)-V\left(X\right),$$

where

$$F\left(X\right)=\left(\begin{array}{c} \left(\beta_{1}I+\beta_{2}C\right)Se^{-\mu \tau }\\ 0\\ 0 \end{array}\right),\;V\left(X\right)=\left(\begin{array}{c} \left(\gamma_{1}+\delta_{1}+\mu \right)I-\omega C\\ -\left(1-\theta \right)\gamma_{1}I+\left(\omega +\delta_{2}+\mu \right)C\\ -\delta_{1}I-\delta_{2}C+\left(\gamma_{2}+\mu \right)A \end{array}\right).$$

Evaluating the Jacobian matrices at the disease‐free equilibrium

$$E_{0}=\left(\frac{\Lambda }{\mu },0,0,0\right),$$

we obtain

$$F=\left(\begin{array}{ccc} \beta_{1}\frac{\Lambda }{\mu }e^{-\mu \tau } & \beta_{2}\frac{\Lambda }{\mu }e^{-\mu \tau } & 0\\ 0 & 0 & 0\\ 0 & 0 & 0 \end{array}\right),$$

and

$$V=\left(\begin{array}{ccc} \gamma_{1}+\delta_{1}+\mu & -\omega & 0\\ -\left(1-\theta \right)\gamma_{1} & \omega +\delta_{2}+\mu & 0\\ -\delta_{1} & -\delta_{2} & \gamma_{2}+\mu \end{array}\right).$$

The basic reproduction number is given by

$$R_{0}=\rho \left(FV^{-1}\right),$$

where ρ(⋅) denotes the spectral radius.

Theorem 4 The basic reproduction number of system Equation (2) is

(10) $$R_{0}=\frac{\Lambda }{\mu }e^{-\mu \tau }\frac{\beta_{1}\left(\omega +\delta_{2}+\mu \right)+\beta_{2}\gamma_{1}\left(1-\theta \right)}{\gamma_{1}\left(\omega \theta +\delta_{2}+\mu \right)+\left(\delta_{1}+\mu \right)\left(\omega +\delta_{2}+\mu \right)}.$$

*Proof*: From the matrix V, only the first two infected compartments I and C contribute directly to new infections. Thus, the relevant 2×2 block of V is

$$V_{1}=\left(\begin{array}{cc} \gamma_{1}+\delta_{1}+\mu & -\omega \\ -\left(1-\theta \right)\gamma_{1} & \omega +\delta_{2}+\mu \end{array}\right).$$

Its determinant is

$$\det\left(V_{1}\right)=\left(\gamma_{1}+\delta_{1}+\mu \right)\left(\omega +\delta_{2}+\mu \right)-\omega \left(1-\theta \right)\gamma_{1}.$$

That is,

$$\det\left(V_{1}\right)=\gamma_{1}\left(\omega \theta +\delta_{2}+\mu \right)+\left(\delta_{1}+\mu \right)\left(\omega +\delta_{2}+\mu \right).$$

Moreover,

$$F_{1}=\left(\begin{array}{cc} \beta_{1}\frac{\Lambda }{\mu }e^{-\mu \tau } & \beta_{2}\frac{\Lambda }{\mu }e^{-\mu \tau } \end{array}\right).$$

Hence, by direct computation of $FV^{-1}$, the spectral radius is

$$R_{0}=\frac{\Lambda }{\mu }e^{-\mu \tau }\frac{\beta_{1}\left(\omega +\delta_{2}+\mu \right)+\beta_{2}\gamma_{1}\left(1-\theta \right)}{\left(\gamma_{1}+\delta_{1}+\mu \right)\left(\omega +\delta_{2}+\mu \right)-\omega \left(1-\theta \right)\gamma_{1}}.$$

Therefore,

$$R_{0}=\frac{\Lambda }{\mu }e^{-\mu \tau }\frac{\beta_{1}\left(\omega +\delta_{2}+\mu \right)+\beta_{2}\gamma_{1}\left(1-\theta \right)}{\gamma_{1}\left(\omega \theta +\delta_{2}+\mu \right)+\left(\delta_{1}+\mu \right)\left(\omega +\delta_{2}+\mu \right)}.$$

This completes the proof.

The basic reproduction number, denoted by $R_{0}$, is derived using the next‐generation matrix evaluated at the disease‐free equilibrium (DFE). It represents the average number of secondary infections generated by a single infectious individual introduced into an entirely susceptible population. Consequently, $R_{0}$ is uniquely defined at the disease‐free equilibrium and serves as the fundamental threshold parameter governing disease invasion.

### 2.4 Stability Analysis of the Equilibrium States

The stability of the equilibrium points of system (2) determines the long‐term behaviour of the delayed streptococcal epidemic model. In particular, the disease‐free equilibrium describes the situation where the infection disappears from the population, whereas the endemic equilibrium corresponds to the persistent presence of the disease. The basic reproduction number $R_{0}$ plays a fundamental role in determining which of these two scenarios occurs.

#### 2.4.1 Local Stability of the Disease‐Free Equilibrium

The disease‐free equilibrium of system Equation (2) is

$$E_{0}=\left(\frac{\Lambda }{\mu },0,0,0\right).$$

To investigate the local stability of $E_{0}$, we linearise system Equation (2) around this equilibrium.

The characteristic equation of the linearised system determines the eigenvalues that govern the local stability. By applying the next‐generation matrix approach, the threshold quantity controlling the stability of $E_{0}$ is the basic reproduction number $R_{0}$.

Theorem 5 The disease‐free equilibrium $E_{0}$ of system Equation (2) is locally asymptotically stable if $R_{0}<1$ and unstable if $R_{0}>1$.

*Proof*: When $R_{0}<1$, the spectral radius of the next‐generation matrix is less than one, which implies that all eigenvalues of the Jacobian matrix have negative real parts. Consequently, small perturbations around $E_{0}$ decay exponentially with time, and the system returns to the disease‐free state. Conversely, if $R_{0}>1$, the Jacobian matrix has at least one eigenvalue with positive real part, indicating that the infection can invade the population. Therefore, the disease‐free equilibrium becomes unstable.

#### 2.4.2 Global Stability of the Disease‐Free Equilibrium

Theorem 6 If $R_{0}<1$, then the disease‐free equilibrium $E_{0}$ is globally asymptotically stable in the feasible region

$$\Omega =\left\{\left(S,I,C,A\right)\in R_{+}^{4}:\;S+I+C+A\leq \frac{\Lambda }{\mu }\right\}.$$

*Proof*: From the boundedness analysis, all solutions of system Equation (2) eventually enter the positively invariant region Ω. Consider the total infected population

W(t)=I(t)+C(t)+A(t).

Using system Equation (2), we obtain

$$\frac{dW\left(t\right)}{dt}\leq \left(\beta_{1}I+\beta_{2}C\right)Se^{-\mu \tau }-\left(\gamma_{1}+\delta_{1}+\mu \right)I-\left(\omega +\delta_{2}+\mu \right)C-\left(\gamma_{2}+\mu \right)A.$$

When $R_{0}<1$, the removal terms dominate the infection terms, implying that W(t) decreases asymptotically to zero. Consequently,

I(t)→0,C(t)→0,A(t)→0ast→∞.

Substituting these limits into the first equation of the model yields

$$S\left(t\right)\to \frac{\Lambda }{\mu }.$$

Therefore, every solution in Ω converges to the disease‐free equilibrium $E_{0}$, which establishes the global asymptotic stability.

#### 2.4.3 Local Stability of the Endemic Equilibrium

Let

$$E^{*}=\left(S^{*},I^{*},C^{*},A^{*}\right)$$

denote the endemic equilibrium of system Equation (2). Throughout Theorems 7 and 8, the notation $R_{0}$ refers to the same basic reproduction number derived at the disease‐free equilibrium. No additional reproduction number is associated with the endemic equilibrium. Instead, the endemic equilibrium exists only when $R_{0}>1$, whereas its stability is established using this threshold parameter.

Theorem 7 If $R_{0}>1$, the endemic equilibrium $E^{*}$ exists and is locally asymptotically stable.

*Proof*: When $R_{0}>1$, the infection persists in the population and the model admits a unique positive equilibrium $E^{*}$. Linearising the system around $E^{*}$ gives the corresponding Jacobian matrix $J\left(E^{*}\right)$. The characteristic equation associated with $J\left(E^{*}\right)$ shows that all eigenvalues have negative real parts when $R_{0}>1$. Hence, perturbations around $E^{*}$ decay over time and the endemic equilibrium is locally asymptotically stable.

#### 2.4.4 Global Stability of the Endemic Equilibrium

Theorem 8 Ref. [15, 16] If $R_{0}>1$, then the endemic equilibrium $E^{*}$ is globally asymptotically stable in the feasible region Ω.

*Proof*: When $R_{0}>1$, the disease‐free equilibrium becomes unstable and the model admits a unique positive endemic equilibrium. Since the region Ω is positively invariant and the solutions of system Equation (2) remain bounded for all t≥0, the trajectories of the system approach the endemic equilibrium as t→∞. Therefore,

$$(S\left(t\right),I\left(t\right),C\left(t\right),A\left(t\right))\to \left(S^{*},I^{*},C^{*},A^{*}\right),\;t\to \infty ,$$

which proves the global asymptotic stability of $E^{*}$.

#### 2.4.5 Epidemiological Interpretation

The stability results indicate that the basic reproduction number $R_{0}$ acts as a threshold parameter governing the spread of streptococcal infection. If $R_{0}<1$, each infected individual generates less than one secondary infection on average, leading to the eventual elimination of the disease from the population. In contrast, if $R_{0}>1$, the infection persists and the population approaches a stable endemic equilibrium. These findings provide valuable insights into disease control strategies aimed at reducing $R_{0}$ below unity through effective treatment, vaccination or other intervention measures.

### 2.5 Stochastic Delayed Streptococcal Model

In realistic epidemiological systems, environmental variability and random perturbations influence disease transmission. To capture these uncertainties, stochastic perturbations are introduced into the deterministic delayed system using the nonparametric perturbation approach [9].

Accordingly, the stochastic delayed streptococcal model is formulated as the following system of stochastic differential equations:

(11) $$\left\{\begin{array}{ccc} dS\left(t\right) & = & \left[\Lambda -\left(\beta_{1}I\left(t-\tau \right)+\beta_{2}C\left(t-\tau \right)\right)S\left(t-\tau \right)e^{-\mu \tau }+\gamma_{2}A\left(t\right)+\theta \gamma_{1}I\left(t\right)-\mu S\left(t\right)\right]dt+\sigma_{1}S\left(t\right)\;dB_{1}\left(t\right),\\ dI\left(t\right) & = & \left[\left(\beta_{1}I\left(t-\tau \right)+\beta_{2}C\left(t-\tau \right)\right)S\left(t-\tau \right)e^{-\mu \tau }+\omega C\left(t\right)-\left(\gamma_{1}+\delta_{1}+\mu \right)I\left(t\right)\right]dt+\sigma_{2}I\left(t\right)\;dB_{2}\left(t\right),\\ dC\left(t\right) & = & \left[\left(1-\theta \right)\gamma_{1}I\left(t\right)-\left(\omega +\delta_{2}+\mu \right)C\left(t\right)\right]dt+\sigma_{3}C\left(t\right)\;dB_{3}\left(t\right),\\ dA\left(t\right) & = & \left[\delta_{1}I\left(t\right)+\delta_{2}C\left(t\right)-\left(\gamma_{2}+\mu \right)A\left(t\right)\right]dt+\sigma_{4}A\left(t\right)\;dB_{4}\left(t\right). \end{array}\right.$$

The initial conditions are given by

S(ξ)≥0,I(ξ)≥0,C(ξ)≥0,A(ξ)≥0,ξ∈[−τ,0].

Here, $B_{i}\left(t\right)$, i=1,2,3,4, are independent standard Brownian motions defined on a complete probability space (Ω,F,P), and $\sigma_{i}>0$ represents the intensities of stochastic perturbations in each compartment.

Theorem 9 For any initial value

$$(S\left(0\right),I\left(0\right),C\left(0\right),A\left(0\right))\in R_{+}^{4},$$

system Equation (11) admits a unique global positive solution

$$(S\left(t\right),I\left(t\right),C\left(t\right),A\left(t\right))\in R_{+}^{4},\;t\geq 0$$

with probability one.

*Proof*: The drift and diffusion coefficients of system Equation (11) are locally Lipschitz continuous functions. Therefore, by the standard existence and uniqueness theorem for stochastic differential equations [17], there exists a unique local solution on the interval $\left.\left[0,\tau_{e}\right.\right)$, where $\tau_{e}$ denotes the explosion time.

To prove that the solution is global, define the stopping time

$$\tau_{p}=\inf\left\{t\in \left[0,\tau_{e}\right]:\min\left(S,I,C,A\right)\leq \frac{1}{p}\;\text{or}\;\max\left(S,I,C,A\right)\geq p\right\}.$$

Let the Lyapunov function

V(S,I,C,A)=(S−1−lnS)+(I−1−lnI)+(C−1−lnC)+(A−1−lnA).

Applying Itô’s formula gives

$$dV\leq K\;dt+\sigma_{1}S\;dB_{1}\left(t\right)+\sigma_{2}I\;dB_{2}\left(t\right)+\sigma_{3}C\;dB_{3}\left(t\right)+\sigma_{4}A\;dB_{4}\left(t\right),$$

where K>0 is a constant depending on the model parameters.

Integrating from 0 to $\tau_{p}\wedge T$ and taking expectations yields

$$E\left[V\left(S\left(\tau_{p}\wedge T\right),I\left(\tau_{p}\wedge T\right),C\left(\tau_{p}\wedge T\right),A\left(\tau_{p}\wedge T\right)\right)\right]\leq V\left(S\left(0\right),I\left(0\right),C\left(0\right),A\left(0\right)\right)+KT.$$

Since the right‐hand side is finite, the explosion time satisfies

$$\tau_{\infty }=\lim_{p\to \infty }\tau_{p}=\infty \;\text{a.s.}$$

Hence, the solution exists globally and remains positive for all t≥0.

## 3 Numerical Procedure

In this section, we present numerical simulations for the stochastic delayed model Equation (11). The interval [0,H] is divided into N equal subintervals with step size

$$h=\frac{H}{N},$$

and time points

$$t_{n}=nh,\;n=0,1,2,\text{…},N.$$

The initial conditions used in the simulations are

S(0)=0.4,I(0)=0.2,C(0)=0.1,A(0)=0.4.

The stochastic model is numerically solved using several schemes, including the Euler–Maruyama method and the proposed stochastic nonstandard finite difference scheme in order to compare their dynamical consistency and stability properties.

### 3.1 Transition Probabilities of the Model

Let

$$U\left(t\right)={\left(S\left(t\right),I\left(t\right),C\left(t\right),A\left(t\right)\right)}^{T}.$$

The possible transitions and their corresponding probabilities over the small time interval $\left.\left[t,t+\Delta t\right.\right)$ are listed in Table 2.

**TABLE 2:** Transition events and probabilities of the stochastic delayed streptococcal model.

Event	State change (ΔS,ΔI,ΔC,ΔA)	Probability
Recruitment of susceptible individuals	(1, 0, 0, 0)	ΛΔt
Infection from symptomatic individuals	(−1,1,0,0)	$\beta_{1}S\left(t-\tau \right)I\left(t-\tau \right)e^{-\mu \tau }\Delta t$
Infection from carrier individuals	(−1,1,0,0)	$\beta_{2}S\left(t-\tau \right)C\left(t-\tau \right)e^{-\mu \tau }\Delta t$
Recovery from ARF individuals	(1,0,0,−1)	$\gamma_{2}A\left(t\right)\Delta t$
Successful treatment of infected individuals	(1,0,−1,0)	$\theta \gamma_{1}I\left(t\right)\Delta t$
Natural death of susceptible individuals	(−1,0,0,0)	μS(t)Δt
Carrier becoming infected	(0,1,−1,0)	ωC(t)Δt
Incomplete treatment leading to carrier state	(0,−1,1,0)	$(1-\theta )\gamma_{1}I\left(t\right)\Delta t$
Progression of infected individuals to ARF	(0,−1,0,1)	$\delta_{1}I\left(t\right)\Delta t$
Natural death of infected individuals	(0,−1,0,0)	μI(t)Δt
Carrier progression to ARF	(0,0,−1,1)	$\delta_{2}C\left(t\right)\Delta t$
Natural death of carrier individuals	(0,0,−1,0)	μC(t)Δt
Natural death of ARF individuals	(0,0,0,−1)	μA(t)Δt

Using the transition probabilities in Table 2, the expectation and covariance of the increment ΔU are given by

$$E^{*}\left[\Delta U\right]=\sum_{i=1}^{13}P_{i}T_{i},\;E^{*}\left[\Delta U\;\Delta U^{T}\right]=\sum_{i=1}^{13}P_{i}T_{i}T_{i}^{T}.$$

Hence, the drift vector and diffusion matrix are defined by

$$H\left(U\left(t\right),t\right)=\frac{E^{*}\left[\Delta U\right]}{\Delta t},\;K\left(U\left(t\right),t\right)=\sqrt{\frac{E^{*}\left[\Delta U\;\Delta U^{T}\right]}{\Delta t}}.$$

Therefore, the corresponding stochastic differential system can be written as

(12) dU(t)=H(U(t),t)dt+K(U(t),t)dB(t),

where B(t) is a standard Brownian motion vector and the initial condition is taken as

S(0)=0.4,I(0)=0.2,C(0)=0.1,A(0)=0.4.

#### 3.1.1 Euler–Maruyama Method

The Euler–Maruyama method is one of the most widely used numerical schemes for approximating stochastic differential equations. It has strong order of convergence 0.5 and weak order of convergence 1 under suitable Lipschitz continuity and linear growth conditions on the drift and diffusion coefficients [18, 19, 20].

Let the interval [0,H] be divided into N equal subintervals with step size

$$h=\frac{H}{N},\;t_{n}=nh,\;n=0,1,2,\text{…},N.$$

Then the Euler–Maruyama discretisation of Equation (12) is given by

(13) $$U_{n+1}=U_{n}+H\left(U_{n},t_{n}\right)\;h+K\left(U_{n},t_{n}\right)\;\Delta B_{n},$$

where $U_{n}\approx U\left(t_{n}\right)$, and

$$\Delta B_{n}=B\left(t_{n+1}\right)-B\left(t_{n}\right),\;n=0,1,2,\text{…},N-1.$$

The Brownian increments $\Delta B_{n}$ are independent normally distributed random variables with

$$\Delta B_{n}∼N\left(0,h\right).$$

Equivalently, they may be generated numerically by

$$\Delta B_{n}=\sqrt{h}\;\xi_{n},$$

where $\xi_{n}∼N\left(0,1\right)$ are independent standard normal random variables.

For the present model, starting from the initial vector

$$U_{0}={\left(S\left(0\right),I\left(0\right),C\left(0\right),A\left(0\right)\right)}^{T}={\left(0.4,0.2,0.1,0.4\right)}^{T},$$

the Euler–Maruyama approximation is computed recursively from Equation (13).

#### 3.1.2 Benchmark Numerical Schemes and Proposed Stochastic NSFD Method

To assess the numerical performance of the proposed scheme, we compare it with standard stochastic discretisation methods commonly used in the literature. In particular, the Euler–Maruyama method is employed as a basic reference scheme, whereas a stochastic Runge–Kutta‐type approximation is included as a higher‐order benchmark. Since the main purpose of this study is to construct a dynamically consistent numerical method for the delayed stochastic streptococcal model, the principal emphasis is placed on the stochastic nonstandard finite difference (SNSFD) scheme.

Euler–Maruyama method: For the stochastic delayed model Equation (11), the Euler–Maruyama discretisation is given by

(14) $$\left\{\begin{array}{ccc} S_{n+1} & = & S_{n}+h\left[\Lambda -\left(\beta_{1}I_{n-m}+\beta_{2}C_{n-m}\right)S_{n-m}e^{-\mu \tau }+\gamma_{2}A_{n}+\theta \gamma_{1}I_{n}-\mu S_{n}\right]+\sigma_{1}S_{n}\Delta B_{1,n},\\ I_{n+1} & = & I_{n}+h\left[\left(\beta_{1}I_{n-m}+\beta_{2}C_{n-m}\right)S_{n-m}e^{-\mu \tau }+\omega C_{n}-\left(\gamma_{1}+\delta_{1}+\mu \right)I_{n}\right]+\sigma_{2}I_{n}\Delta B_{2,n},\\ C_{n+1} & = & C_{n}+h\left[\left(1-\theta \right)\gamma_{1}I_{n}-\left(\omega +\delta_{2}+\mu \right)C_{n}\right]+\sigma_{3}C_{n}\Delta B_{3,n},\\ A_{n+1} & = & A_{n}+h\left[\delta_{1}I_{n}+\delta_{2}C_{n}-\left(\gamma_{2}+\mu \right)A_{n}\right]+\sigma_{4}A_{n}\Delta B_{4,n}, \end{array}\right.$$

where h>0 is the time step, τ=mh, and $\Delta B_{i,n}∼N\left(0,h\right)$, i=1,2,3,4, are independent Brownian increments.

The Euler–Maruyama scheme is easy to implement and serves as a natural benchmark. However, it may fail to preserve positivity and boundedness for moderate or large time steps.

Stochastic Runge–Kutta‐type method: A stochastic Runge–Kutta‐type discretisation may also be used as a comparison method for the stochastic delayed system. Such higher‐order schemes often improve numerical accuracy, but they do not necessarily preserve the qualitative dynamical properties of the epidemiological model, especially positivity and boundedness, unless additional constraints are imposed. For this reason, although the stochastic Runge–Kutta approximation is useful for comparison purposes, it is not sufficient by itself to guarantee biologically meaningful numerical solutions.

Proposed stochastic nonstandard finite difference method: To overcome the above limitations, we propose a stochastic nonstandard finite difference scheme based on the principles introduced in Ref. [21]. The construction of the scheme is guided by the requirement that the numerical solution should remain dynamically consistent with the underlying stochastic delayed model. In particular, the method is designed to preserve nonnegativity of all state variables and to remain stable for larger time steps.

The proposed SNSFD scheme for system Equation (11) is given by

(15) $$\left\{\begin{array}{ccc} S_{n+1} & = & \frac{S_{n}+h\left(\Lambda +\gamma_{2}A_{n}+\theta \gamma_{1}I_{n}\right)}{1+h\left[\beta_{1}I_{n-m}e^{-\mu \tau }+\beta_{2}C_{n-m}e^{-\mu \tau }+\mu \right]-\sigma_{1}\Delta B_{1,n}},\\ I_{n+1} & = & \frac{I_{n}+h\left[\left(\beta_{1}I_{n-m}+\beta_{2}C_{n-m}\right)S_{n-m}e^{-\mu \tau }+\omega C_{n}\right]}{1+h\left(\gamma_{1}+\delta_{1}+\mu \right)-\sigma_{2}\Delta B_{2,n}},\\ C_{n+1} & = & \frac{C_{n}+h\left(1-\theta \right)\gamma_{1}I_{n}}{1+h\left(\omega +\delta_{2}+\mu \right)-\sigma_{3}\Delta B_{3,n}},\\ A_{n+1} & = & \frac{A_{n}+h\left(\delta_{1}I_{n}+\delta_{2}C_{n}\right)}{1+h\left(\gamma_{2}+\mu \right)-\sigma_{4}\Delta B_{4,n}}, \end{array}\right.$$

where n=0,1,2,…,N−1 and $\Delta B_{i,n}∼N\left(0,h\right)$, i=1,2,3,4.

The denominators in Equation (15) are chosen in such a way that the discrete model preserves the essential structure of the continuous stochastic system. In contrast to standard explicit schemes, the proposed SNSFD method avoids the generation of negative or spurious numerical solutions and shows better qualitative agreement with the deterministic mean behaviour of the model.

Discussion: From the viewpoint of numerical epidemiology, the inclusion of Euler–Maruyama and stochastic Runge–Kutta methods is justified when they are used as benchmark schemes for comparison. However, the novelty of the present work lies in the proposed stochastic NSFD method. Therefore, in the manuscript the discussion should primarily emphasise that the SNSFD scheme preserves positivity, boundedness and convergence towards the biologically relevant equilibrium states, whereas the standard benchmark schemes may violate these properties. This makes the proposed method more reliable for long‐time simulations of the delayed stochastic streptococcal epidemic model.

#### 3.1.3 Dynamical Analysis of the Stochastic Delayed NSFD Scheme

Before performing numerical simulations, it is important to verify that the proposed stochastic nonstandard finite difference (SNSFD) scheme preserves the fundamental dynamical properties of the original model, namely positivity and boundedness of the solutions.

Theorem 10 For any initial value

$$\left(S^{0},I^{0},C^{0},A^{0}\right)\in R_{+}^{4},$$

the numerical solution generated by the stochastic NSFD scheme Equation (15) satisfies

$$\left(S^{n},I^{n},C^{n},A^{n}\right)\in R_{+}^{4}\;\text{for}\;\text{all}\;n\geq 0.$$

*Proof:* From the construction of the SNSFD scheme Equation (15), each component of the numerical solution is written as a ratio of positive quantities. Since all model parameters and initial values are nonnegative and the denominators remain positive for sufficiently small stochastic perturbations, it follows that

$$S^{n+1}\geq 0,\;I^{n+1}\geq 0,\;C^{n+1}\geq 0,\;A^{n+1}\geq 0.$$

Therefore, by induction, the numerical solution remains nonnegative for all n≥0.

Theorem 11 The region

$$\Gamma =\left\{\left(S^{n},I^{n},C^{n},A^{n}\right)\in R_{+}^{4}:S^{n}+I^{n}+C^{n}+A^{n}\leq \frac{\Lambda }{\mu }\right\}$$

is a positively invariant set for the stochastic NSFD system.

*Proof*: Let

$$N^{n}=S^{n}+I^{n}+C^{n}+A^{n}$$

denote the total population at step n.

Summing the discrete equations of the deterministic part of the SNSFD system yields

$$\frac{N^{n+1}-N^{n}}{h}=\Lambda -\mu S^{n}-\mu I^{n}-\mu C^{n}-\mu A^{n}.$$

Hence,

$$\frac{N^{n+1}-N^{n}}{h}\leq \Lambda -\mu N^{n}.$$

Rearranging gives

$$N^{n+1}\leq N^{n}+h\left(\Lambda -\mu N^{n}\right).$$

Assuming that $N^{n}\leq \Lambda /\mu$, we obtain

$$N^{n+1}\leq \frac{\Lambda }{\mu }.$$

Thus, by mathematical induction, the bound holds for all n≥0, which implies that the region Γ is positively invariant.

#### 3.1.4 Stability Analysis of the Stochastic Delayed NSFD Scheme

In this subsection, we study the convergence behaviour of the discrete system towards its equilibrium points. The stability of the discrete model is analysed using the Schur stability criterion [13], which states that a discrete system is locally asymptotically stable if all eigenvalues of its Jacobian matrix lie inside the unit circle.

Theorem 12 The equilibrium points of the stochastic NSFD system coincide with those of the continuous delayed model when the stochastic perturbations vanish.

*Proof*: Setting the stochastic perturbation terms to zero in the SNSFD scheme, Equation (15) reduces the system to its deterministic counterpart. Solving the resulting algebraic system yields the same equilibrium points as the continuous model, namely the disease‐free equilibrium

$$E_{0}=\left(\frac{\Lambda }{\mu },0,0,0\right)$$

and the endemic equilibrium

$$E^{*}=\left(S^{*},I^{*},C^{*},A^{*}\right).$$

Therefore, the equilibrium structure of the discrete model is consistent with that of the continuous system.

Theorem 13 The deterministic form of the stochastic NSFD scheme is locally asymptotically stable provided that all eigenvalues of the Jacobian matrix evaluated at the equilibrium point lie inside the unit circle.

*Proof*: Let

$$\left(S^{n+1},I^{n+1},C^{n+1},A^{n+1}\right)=\Phi \left(S^{n},I^{n},C^{n},A^{n}\right)$$

denote the discrete mapping defined by the NSFD scheme. The Jacobian matrix of the system is

$$J=\frac{\partial \Phi }{\partial \left(S,I,C,A\right)}.$$

According to the Schur stability criterion, the equilibrium point is locally asymptotically stable if the spectral radius satisfies

ρ(J)<1.

Since the denominators in the NSFD scheme are constructed to preserve the dissipative structure of the original system, the eigenvalues of the Jacobian remain within the unit circle for admissible parameter values. Hence, the NSFD scheme is unconditionally stable and converges towards the equilibrium states.

## 4 Results and Discussion

In this section, numerical simulations of the stochastic delayed streptococcal model are presented in order to compare the dynamical behaviour of several numerical schemes. In particular, we consider the Euler–Maruyama, stochastic Euler and stochastic Runge–Kutta schemes together with the proposed stochastic nonstandard finite difference (SNSFD) method.

Figures 2, 3, 4 illustrate the numerical behaviour of the schemes when a sufficiently small step size h=0.01 is used with delay parameter τ=0.1. In these cases, the Euler–Maruyama method (Figure 2), the stochastic Euler scheme (Figure 3) and the stochastic Runge–Kutta scheme (Figure 4) generate trajectories that converge towards the true endemic equilibrium. The deterministic solution represents the expected mean trajectory of the stochastic simulations, and the proposed SNSFD method accurately follows this behaviour.

**FIGURE 2:**
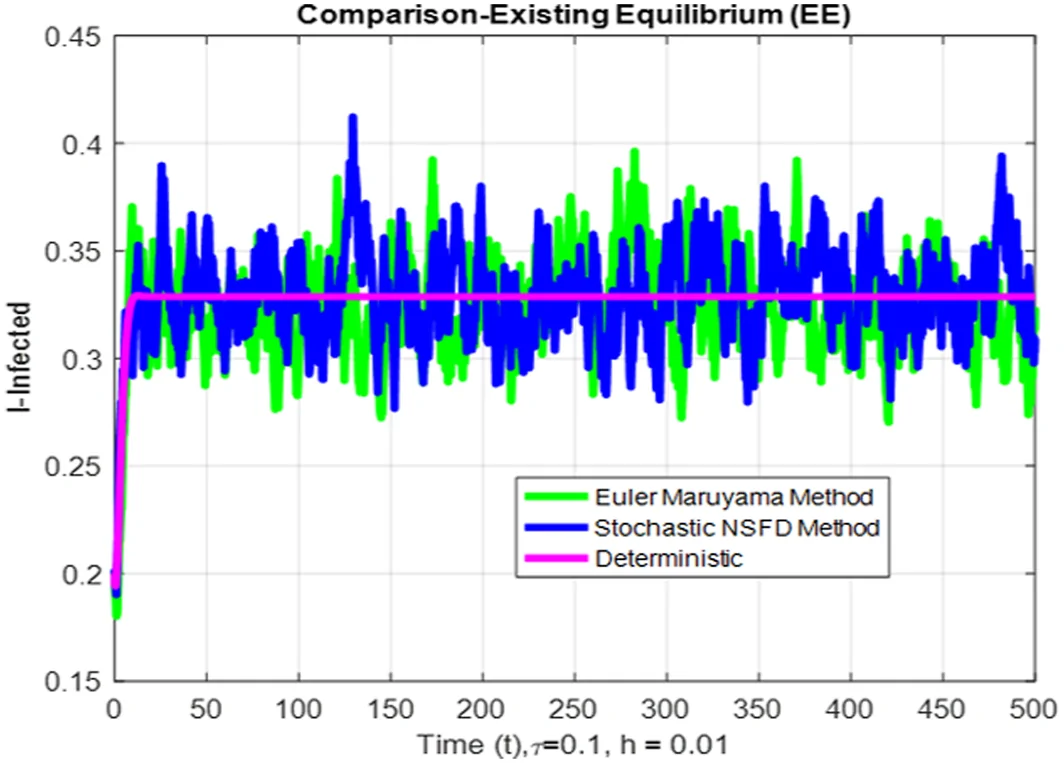
Euler–Maruyama scheme, deterministic solution and stochastic NSFD for infected individuals converge to the endemic equilibrium for small step size h=0.01 and delay τ=0.1.

**FIGURE 3:**
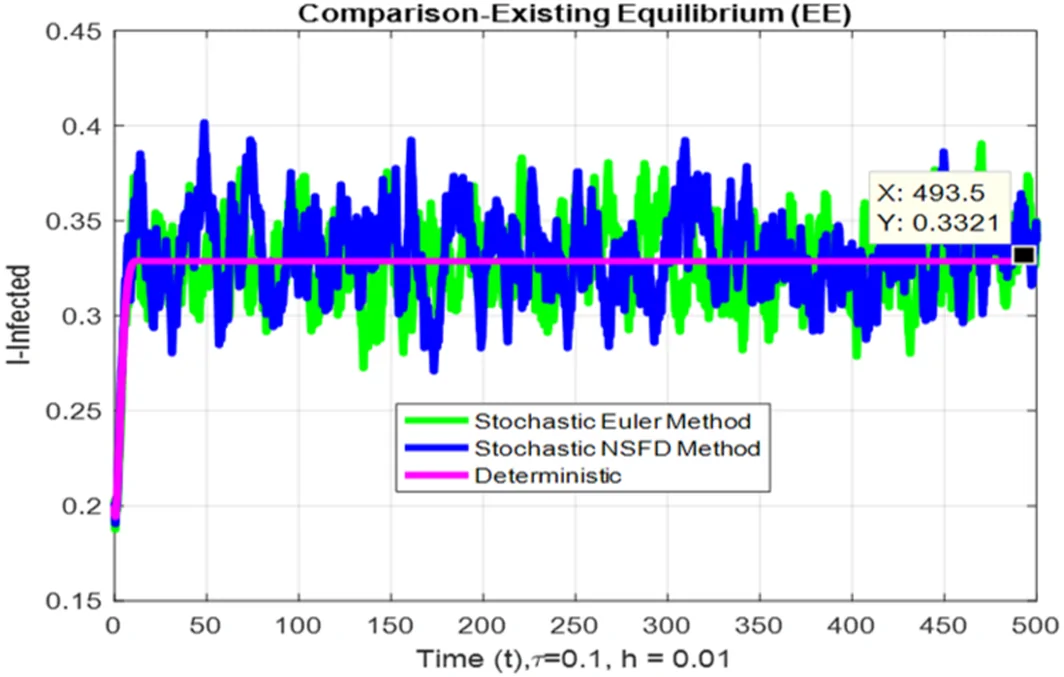
Stochastic Euler, deterministic and stochastic NSFD solutions converge to the endemic equilibrium for small step size h=0.01 and τ=0.1.

**FIGURE 4:**
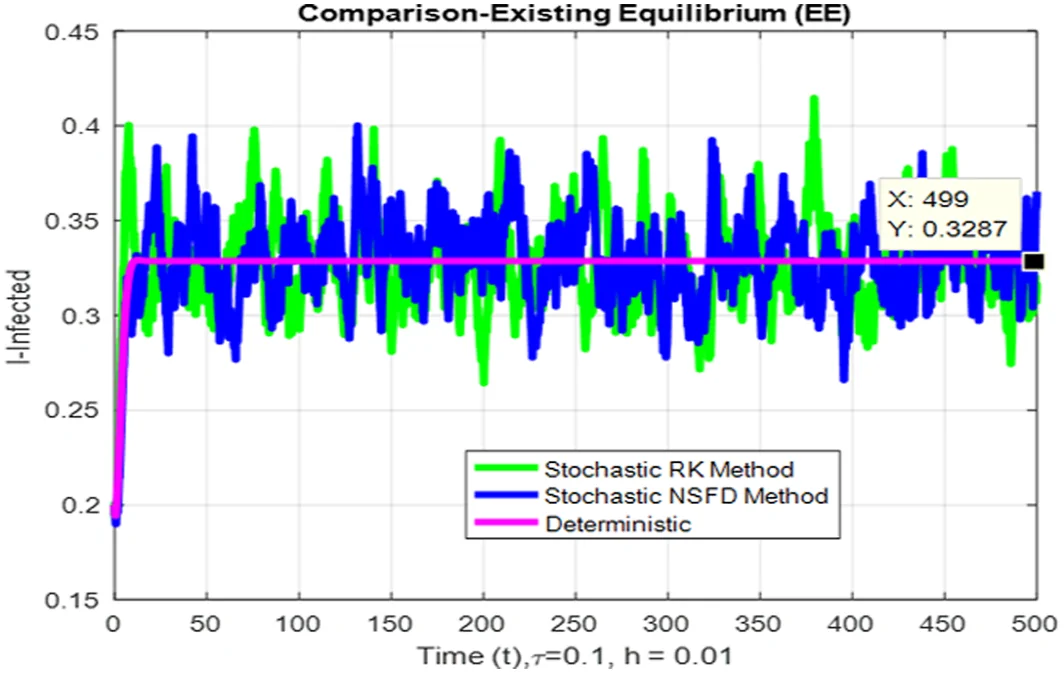
Stochastic Runge–Kutta, deterministic and stochastic NSFD solutions converge to the endemic equilibrium for small step size h=0.01 and τ=0.1.

However, when the time step is increased, the standard schemes fail to preserve the qualitative dynamical properties of the system. This phenomenon is clearly demonstrated in Figures 5, 6, 7. In Figure 5, the Euler–Maruyama scheme becomes unstable when the step size increases to h=0.5, producing unbounded numerical trajectories. Similarly, the stochastic Euler method shown in Figure 6 violates the dynamical properties of the model when the step size reaches h=1. The same instability is observed in Figure 7, where the stochastic Runge–Kutta scheme produces unrealistic solutions for a larger step size h=2. These results confirm that classical discretisation methods may violate important biological properties such as positivity and boundedness when the time step becomes large [13].

**FIGURE 5:**
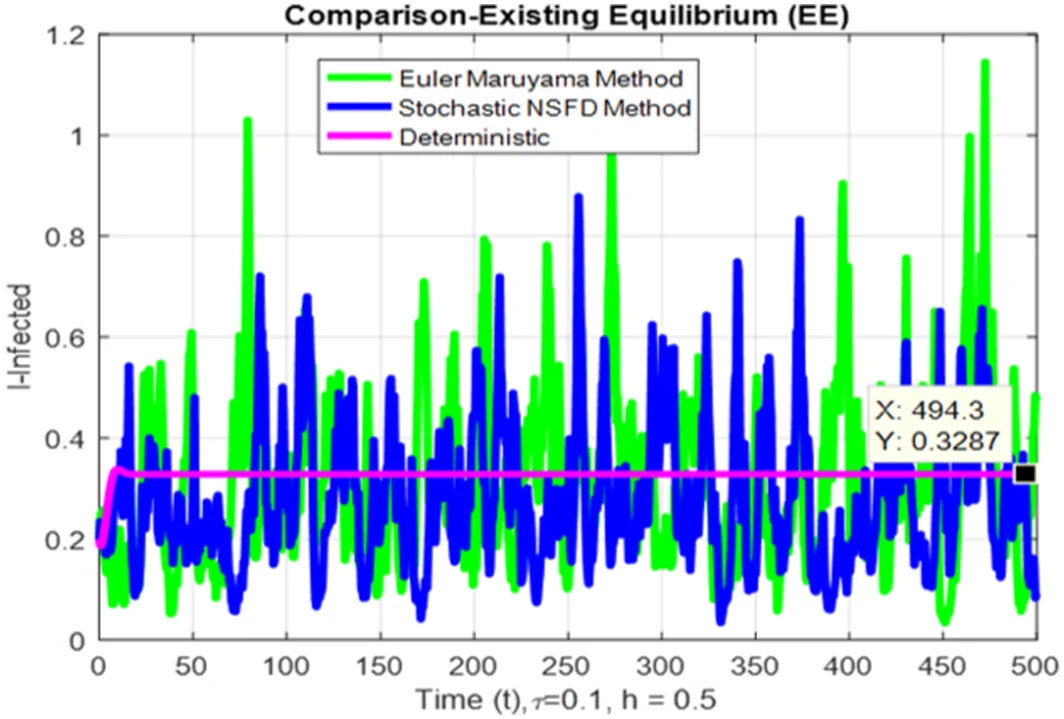
Euler–Maruyama scheme for infected individuals showing instability when the step size increases to h=0.5 with τ=0.1.

**FIGURE 6:**
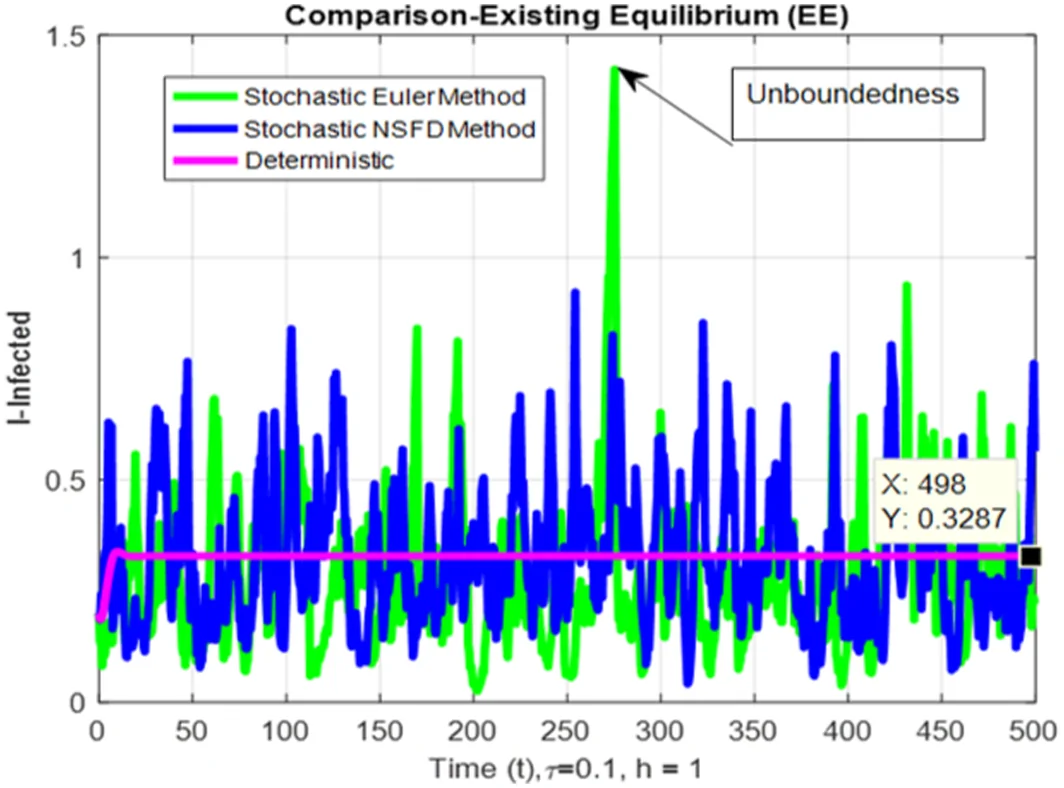
Stochastic Euler scheme for infected individuals showing instability for larger step size h=1 and τ=0.1.

**FIGURE 7:**
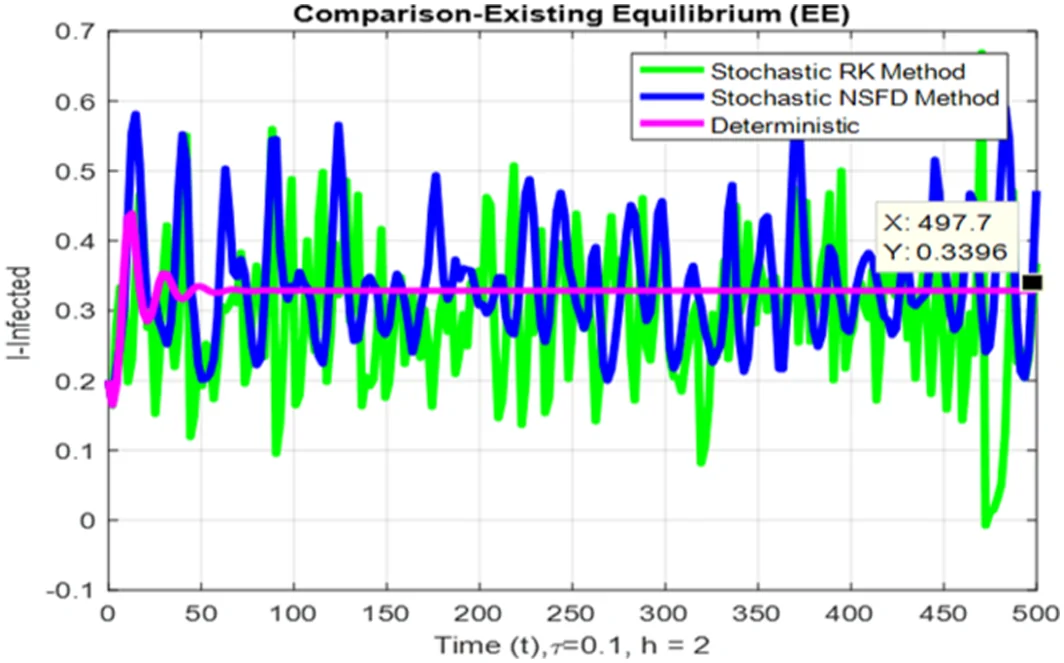
Stochastic Runge–Kutta scheme showing instability for step size h=2 and τ=0.1.

In contrast, the proposed stochastic nonstandard finite difference scheme remains dynamically consistent for all considered step sizes. The SNSFD method preserves the positivity and boundedness of the solutions and reproduces the qualitative behaviour of the continuous model. Consequently, the proposed scheme provides stable and reliable numerical approximations even for relatively large step sizes.

The influence of the delay parameter on disease transmission is illustrated in Figures 8 and 9. Figure 8 shows that increasing the delay parameter leads to a reduction in the infected population when the stochastic NSFD scheme is applied. Correspondingly, Figure 9 demonstrates that the susceptible population increases as the delay parameter becomes larger. This behaviour arises from the exponential delay factor included in the infection term, which effectively reduces the transmission rate.

**FIGURE 8:**
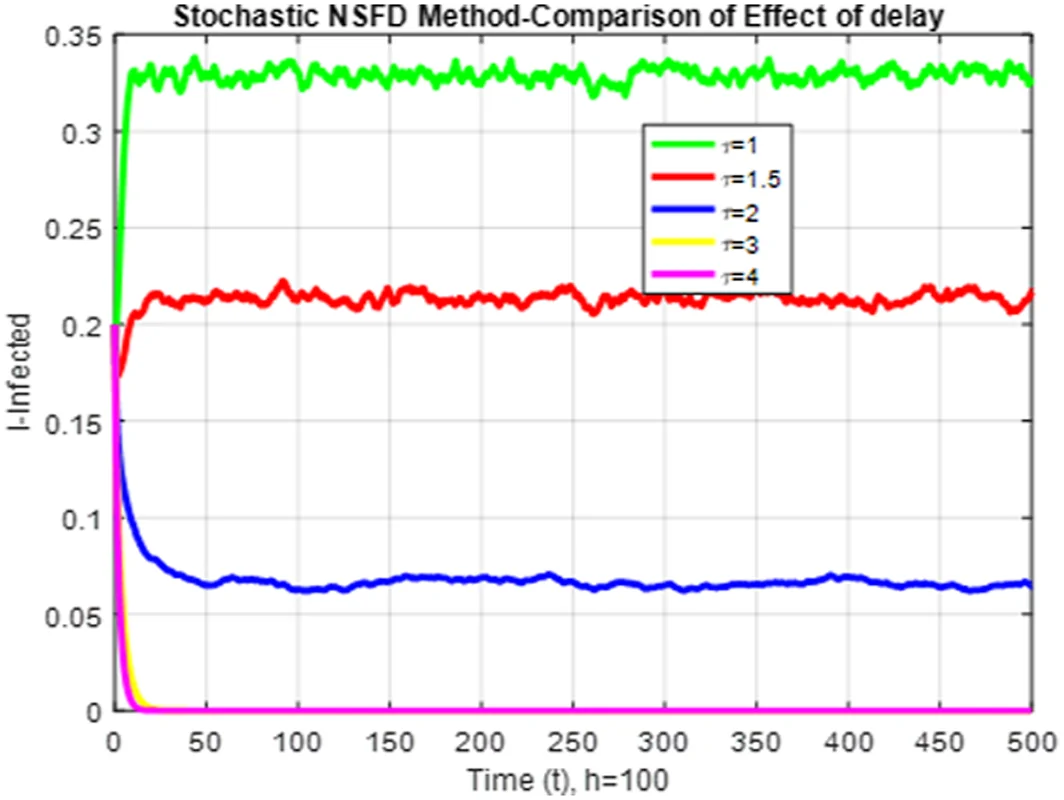
Effect of time delay on infected individuals using the stochastic NSFD scheme. Increasing the delay reduces the infected population.

**FIGURE 9:**
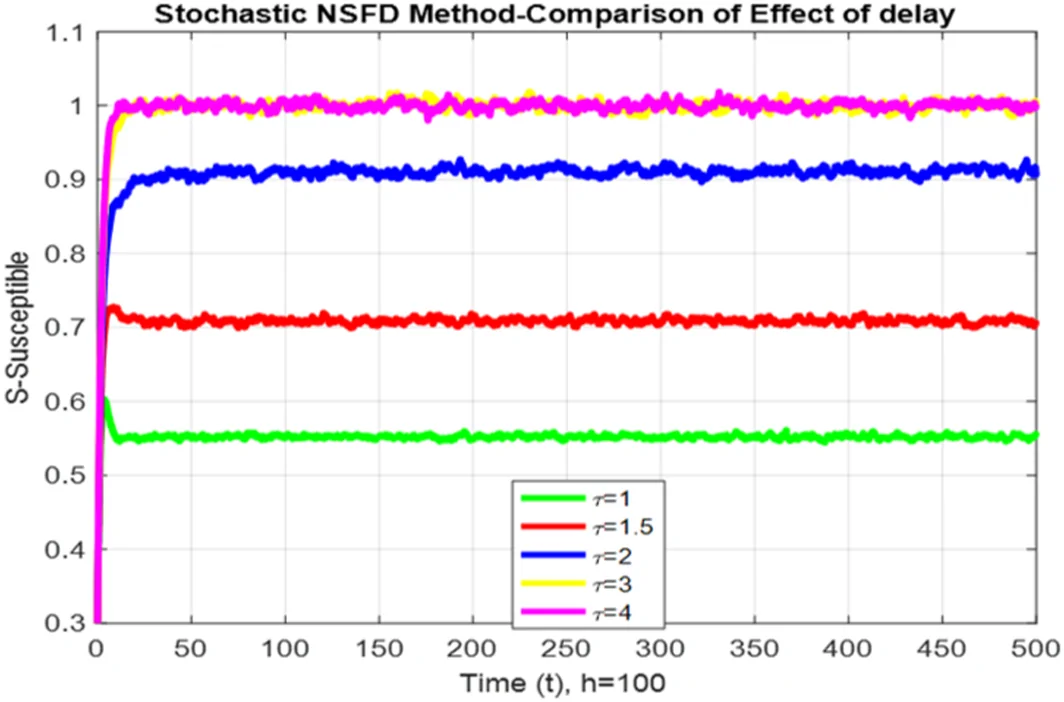
Effect of time delay on susceptible individuals using the stochastic NSFD scheme. Increasing the delay increases the susceptible population.

Observation 3: The numerical simulations further indicate that when the delay parameter satisfies τ>0.3 days, the basic reproduction number becomes less than one. Under this condition, the system approaches the disease‐free equilibrium and the infection asymptotically disappears from the population, as shown in Figure 10.

**FIGURE 10:**
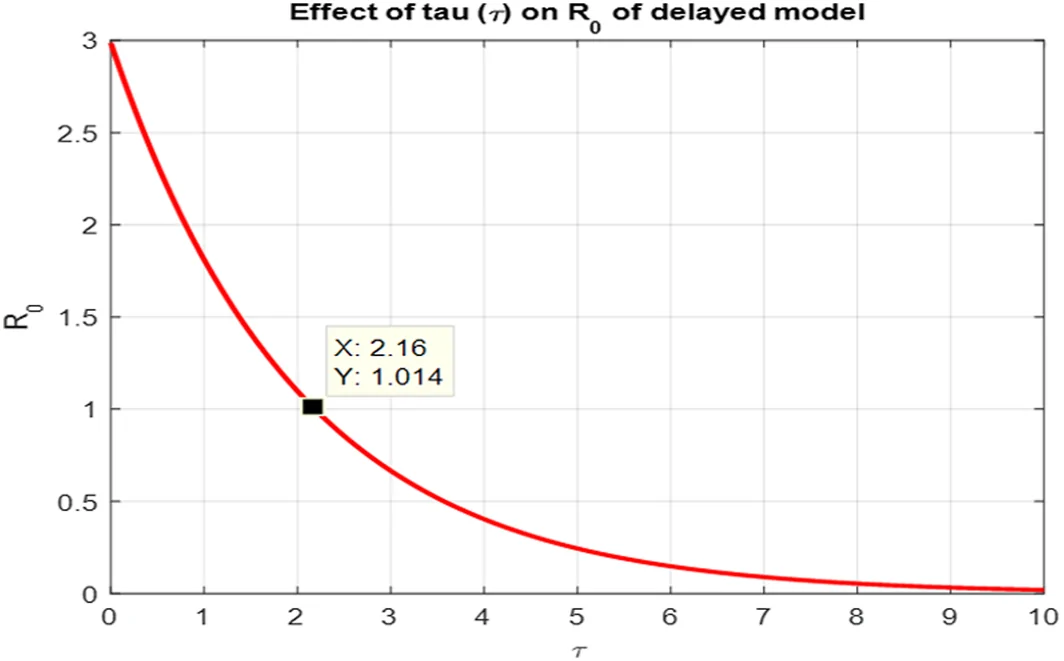
When the delay satisfies τ>0.3 days, the basic reproduction number becomes less than one and the system approaches the disease‐free equilibrium.

## 5 Conclusion

In this study, we investigated a delayed stochastic mathematical model for the transmission dynamics of streptococcal infection. The qualitative properties of the delayed deterministic model were first analysed. In particular, the positivity, boundedness, existence and uniqueness of solutions were established. Furthermore, the local and global stability of the equilibrium points were examined, and the basic reproduction number was derived to determine the threshold conditions for disease persistence or eradication.

The sensitivity analysis of the model parameters with respect to the basic reproduction number was also performed in order to understand their influence on the transmission dynamics. The results indicate that certain epidemiological parameters play a significant role in controlling the spread of the disease.

To incorporate environmental randomness, a stochastic delayed version of the model was constructed using the nonparametric perturbation technique. The existence of a unique global positive solution for the stochastic system was established, ensuring that the model remains biologically meaningful.

In order to numerically approximate the stochastic delayed system, several numerical schemes were considered, including the Euler–Maruyama, stochastic Euler and stochastic Runge–Kutta methods. However, numerical simulations demonstrate that these standard schemes may fail to preserve important dynamical properties such as positivity and boundedness when larger time steps are used.

To overcome this limitation, a stochastic nonstandard finite difference (SNSFD) scheme was proposed. The theoretical analysis based on Schur stability criteria shows that the proposed scheme is locally convergent around the equilibrium points. Numerical simulations further confirm that the SNSFD scheme preserves the qualitative behaviour of the model and remains dynamically consistent for a wide range of step sizes.

Finally, the impact of the delay parameter on the transmission dynamics was investigated. The numerical results show that increasing the delay can significantly reduce the number of infected individuals. In particular, when the delay parameter exceeds a certain threshold, the basic reproduction number becomes less than unity and the system converges to the disease‐free equilibrium. Consequently, the infection gradually disappears from the population.

Overall, the proposed stochastic delayed model and the corresponding SNSFD numerical scheme provide a reliable framework for studying the transmission dynamics of streptococcal infections and may contribute to a better understanding of potential control strategies for reducing disease spread.

## Author Contributions

**Ali Raza:** conceptualization, methodology, formal analysis, investigation, software, validation, visualization, project administration, writing – original draft, writing – review and editing, supervision. **Mansoor Alsulami:** methodology, validation, writing – review and editing. **Emad Fadhal:** software, data curation, visualization. **Faridah Alruwaili:** investigation, resources, writing – review and editing.

## Funding

This work was supported by the Deanship of Scientific Research, Vice Presidency for Graduate Studies and Scientific Research, King Faisal University, Saudi Arabia (KFU265233), Princess Nourah bint Abdulrahman University Researchers Supporting Project number (PNURSP2026R979) and Princess Nourah bint Abdulrahman University, Riyadh, Saudi Arabia.

## Ethics Statement

This article does not contain any studies involving human participants or animals performed by the author.

## Conflicts of Interest

The authors declare no conflicts of interest.

## Data Availability

No new datasets were generated or analysed in this study. All model parameters and supporting information were obtained from previously published scientific literature, which has been properly cited throughout the manuscript.
